# Application of Transmission Raman Spectroscopy in Combination with Partial Least-Squares (PLS) for the Fast Quantification of Paracetamol

**DOI:** 10.3390/molecules27051707

**Published:** 2022-03-05

**Authors:** Xuejia Zhao, Ning Wang, Minghui Zhu, Xiaodan Qiu, Shengnan Sun, Yitong Liu, Ting Zhao, Jing Yao, Guangzhi Shan

**Affiliations:** 1 Institute of Medicinal Biotechnology, Chinese Academy of Medical Sciences & Peking Union Medical College, No. 1, Tian Tan Xi Li, Beijing 100050, China; zhaoxuejiajia@163.com (X.Z.); zhuminghui1100@163.com (M.Z.); xdqsdu@163.com (X.Q.); zpsunshengnan@163.com (S.S.); liuyt8210@163.com (Y.L.); zhaoting@imb.pumc.edu.cn (T.Z.); 2 College of Life Science and Technology, Beijing University of Chemical Technology, North Third Ring Road 15, Beijing 100029, China; warning75@163.com; 3 China National Institutes for Food and Drug Control, No. 2, Tian Tan Xi Li, Beijing 100050, China

**Keywords:** transmission Raman spectroscopy, paracetamol, quantitation, chemometrics

## Abstract

In recent years, transmission Raman spectroscopy (TRS) has emerged as a potent new tool for rapid, nondestructive quantitation in pharmaceutical manufacturing. In order to expand the applicability of TRS and enhance its use in product quality monitoring during drug production, we aimed, in the present study, to apply partial least-squares (PLS) approaches to build a model consisting of 150 handmade tablets and covering 15 levels through the use of a multifactor orthogonal design of experiment (DOE), which was used to predict concentrations of validation tablets made by hand. The difference between results according to HPLC and TRS were negligible. The model was used to predict the active pharmaceutical ingredient (API) content in four random commercial paracetamol tablets, and corrected with the spectra of the commercial tablets to obtain four corresponding models. The results show that the content relative error in the model’s predictions after correction with commercially available tablets was significantly lower than that before correction. The corrected model was used to make predictions for 20 tablets from the brand Panadol. Compared with the HPLC results, the prediction relative error was basically less than 4.00%, and the relative standard deviation (RSD) of the content was 0.86%.

## 1. Introduction

In pharmaceutical manufacturing and finished product testing, determining the content of drugs using high-performance liquid chromatography (HPLC) testing is not only time-consuming but also destructive. In recent years, transmission Raman spectroscopy (TRS) has been widely used in the quantification of API and excipients in drugs [1,2,3] and the quantification of polymorphs in pharmaceutical formulations [4,5]. It is a fast and practical technique and also has the ability to obtain highly chemical-specific information and quantitative volumetric data from thick and highly turbid samples [6,7,8].

Compared to HPLC, TRS has the characteristics of no preprocessing, no damage to the sample, and fast determination [9]. In addition, unlike the backscatter mode, transmission Raman geometry can reduce the difference between the results of TRS and HPLC, offering a much improved accuracy and precision by maximizing the sampling volume when the laser beam is directed onto the sample from one side and the Raman signal is collected from the other side, allowing the laser photons to move through the entire body of the sample to convey molecular spectroscopic information on its volumetric content [10,11,12,13].

An additional benefit of this method over conventional backscattering Raman spectroscopy is the ability to suppress Raman and fluorescence signals from a tablet coating or capsule shell [14,15]. TRS typically exhibits excellent specificity with many sharp and distinct features that can be assigned readily to individual components [16], while near-infra-red spectroscopy (NIRS) results often contain broader and overlapping features [17,18]. This makes it easier to interpret TRS results and visualize changes in composition. 

TRS spectra contain multiple peaks of the various Raman-active compounds in a sample, which overlap with each other to form a starting point for a complex, informative analysis. Chemometrics (multivariate analysis) allows us to analyze these complex data. The main multivariate analysis methods are partial least-squares (PLS), principal component analysis (PCA), partial least-squares discriminant analysis (PLS-DA), and constrained regularization (CR) [19]. Compared to other methods, partial least-squares (PLS) is a multivariate data analysis method based on principal component analysis and principal component regression. It is one of the most widely used multivariable calibration methods; it has good selectivity and prediction accuracy, and is suitable for complex multicomponent spectra. PLS can eliminate the influence of data collinearity and effectively reduce the dimensions of spectral data.

Paracetamol is a nonsteroidal antipyretic and analgesic mainly used to treat fever, headache, joint pain, and other symptoms caused by the common cold or influenza [20]. Currently, in the manufacturing of paracetamol tablets and the quantitation of the final product, HPLC is usually used to measure the API content, presenting disadvantages such as the consumption of the chromatographic column and solvent, complicated preprocessing, and deviations in results obtained by different operators. In addition, electroanalytical [21], capillary electrophoretic [22,23], and spectrophotometric methods [24] have also been applied to the determination of paracetamol content [25]. 

The use of TRS for quantification has been previously reported [26,27]; for example, Griffen et al. [1] studied the quantification of all the constituents in a set of tablets consisting of five components (containing paracetamol) using this method. Their study was a proof-of-concept study, which provided sufficient theoretical support for our experiments. The authors demonstrated the feasibility of the technology using a compound handmade tablet, but commercially available tablets are often not suitable for evaluation with the established model due to changes in the composition, proportion, and shape of the tablets. Additionally the study of modeling process parameters such as acquisition time, laser power, and wavelength has not been optimized. In this study, we made use of the spectra of the commercially available tablets to correct the established model, which made the model more applicable and reduced the time required for modeling so that it could be used for high-throughput overall analysis, realizing online batch quality control and nondestructive analysis of continuous production processes.

**Figure 1:**
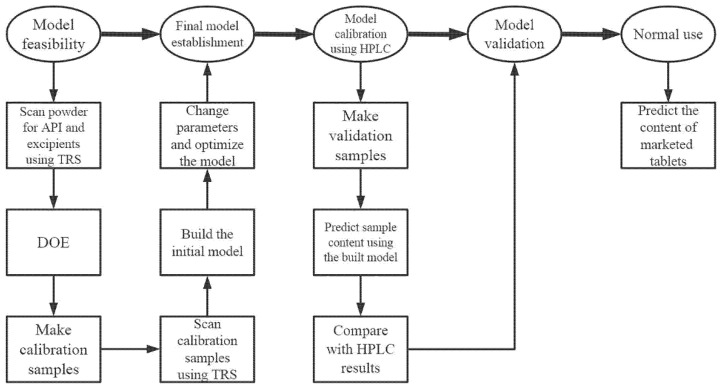
The process of the study.

The purpose of this research was to develop a method for determining the content of paracetamol tablets using transmission Raman spectroscopy in combination with PLS. The model was optimized by changing the type of signal collector, wavelength, preprocessing method, and other parameters, and was corrected by HPLC in order to predict the contents of paracetamol tablets. The API contents in currently marketed paracetamol tablets were predicted and measured and the results were compared with the HPLC results, with the comparison suggesting that the model can be used in pharmaceutical production processes. The specific process is shown in the Figure 1 below.

## 2. Results and Discussion

### 2.1. Method Development

#### 2.1.1. Method Feasibility

In the feasibility stage, we assessed the viability of a TRS application without venturing into a complete method development effort; we compared the API and excipients using a Principal Component Analysis (PCA) dose–response analysis or other appropriate assessment of method feasibility [28,29]. The powdered material of the API and mixture of excipients were dispensed into small, clear 7.5 cm^2^ plastic bags and scanned by TRS with the best acquisition parameters. Because of the large quantity of powder and the high volumetric sensing capability of TRS, the signal contribution of the thin plastic bag here can be considered negligible.

**Figure 2:**
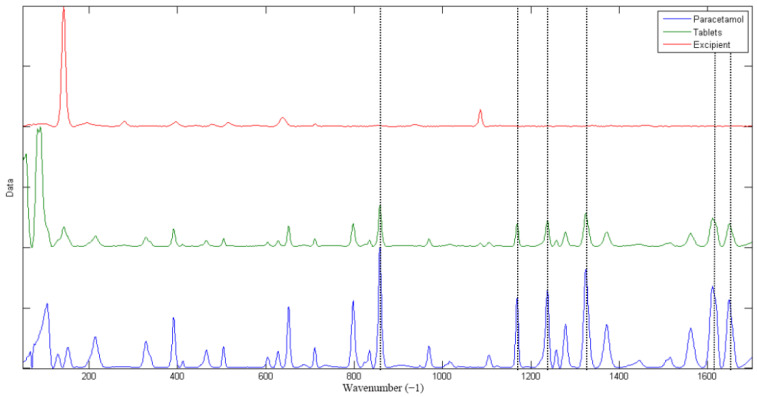
TRS spectra of the samples.

The original spectrum in Figure 2 shows that paracetamol has unique characteristic absorption peaks at 840 cm^−1^, 1170 cm^−1^, 1240 cm^−1^, 1324 cm^−1^, and 1550–1670 cm^−1^, compared to the other components. The peaks at 840 cm^−1^ and 1550–1670 cm^−1^ originated from out-of-plane C-H bending and amide I and amide II bands, respectively [30]. The peaks at 1170 cm^−1^, 1240 cm^−1^, and 1324 cm^−1^ were separately derived from the symmetric stretching vibration of C-N-C, the stretching vibration of benzene –OH, and the symmetric variant of CH_3_. This was recognized by TRS after mixing with other substances so that TRS could be used to quantify the API values of paracetamol tablets.

#### 2.1.2. Development of PLS Calibration Model

PLS is a calibration algorithm, namely a kind of multivariate analysis (MVA) method used for the analysis of mixtures [31]. In the development of the PLS calibration model, seven levels were selected for calibration (* in Table 1). In this study, we explored acquisition parameters such as the type of signal collector, laser power, acquisition time, preprocessing method, and so on, in order to build the most suitable model by assessment of the root mean square error of correction (RMSEC), root mean square error of cross-validation (RMSECV), root mean square error of prediction (RMSEP), and linearity (R^2^) of each model, and made good use of the PLS method to process and analyze the raw data. In a suitable calibration model, there should be no significant differences between RMSEC and RMSECV; if such differences are present, it means the sample is not representative or the model information is not sufficiently extracted. RMSEP was used as the model evaluation index to evaluate the accuracy of prediction. The most suitable models were selected because they combined a low RMSECV and RMSEC with good linearity.

**Figure 3:**
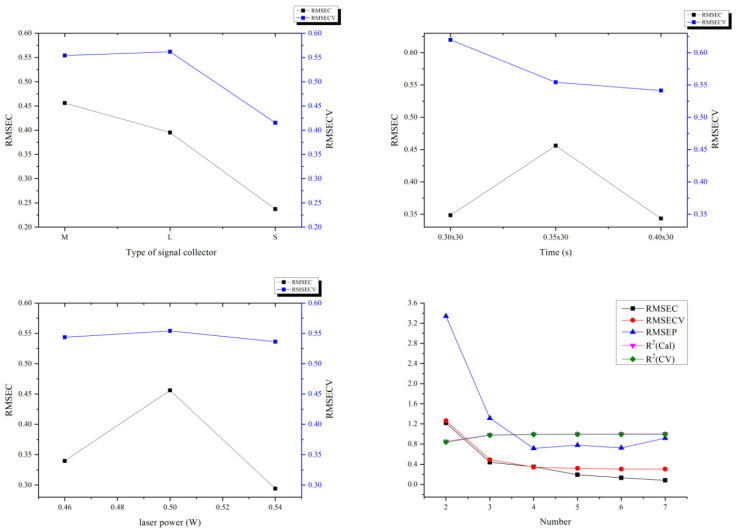
Comparison of the type of signal collector, acquisition time, laser power, and latent variable number of models.

The quality of the model has an important relationship with the instrument parameters and data processing methods. Comparing the results from the varying acquisition parameters, as shown in Figure 3, we can conclude that the model with a 4 mm laser illumination spot diameter at 0.5 w laser power with an M-type signal collector for 10.5 s (0.35 s × 30 accumulations) acquisition time was most suitable, and the optimum processing method for spectra was derivative, multiplicative scatter correction, and mean center (DMM) instead of baselined, standard normal variate, and mean center (BSM) (date not shown). In order to make the model more available, we increased the number of levels from 7 to 15 (all levels in Table 1) and changed the tablet number at each level from 5 to 10 (data not shown). Additionally, we selected the latent variable number 3 instead of 4 to avoid interference by other non-characteristic peaks, although the RMSEC and RMSECV values associated with the number 4 were more closed.

**Figure 4:**
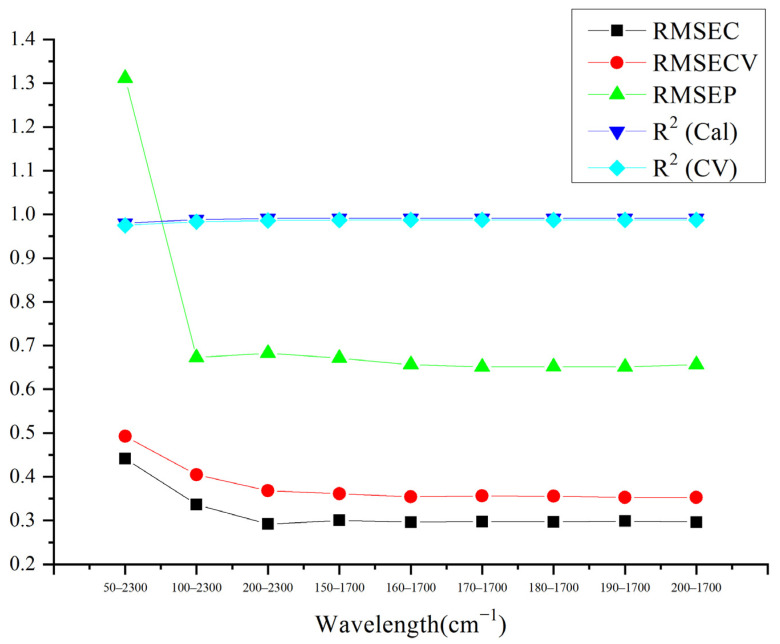
Comparison of the acquisition wavelengths of models.

As shown in Figure 4, when the acquisition wavelength was 1700–170 cm^−1^, the RMSEP was the lowest and the RMSEC and RMSECV were the closest with no significant difference, so that the model had a good prediction accuracy. Furthermore, the excipients had a very distinctive peak at about 180 cm^−1^ (as shown in Figure 2), which the acquisition wavelength range should contain, so that the relative intensities of the API and excipient signals could be compared during the modeling process.

In order to make the results predicted by the model closer to the true values, the API theoretical concentration (in Table 1) in the original model was replaced with the actual concentration measured by HPLC and the model was corrected after optimizing parameters.

**Figure 5:**
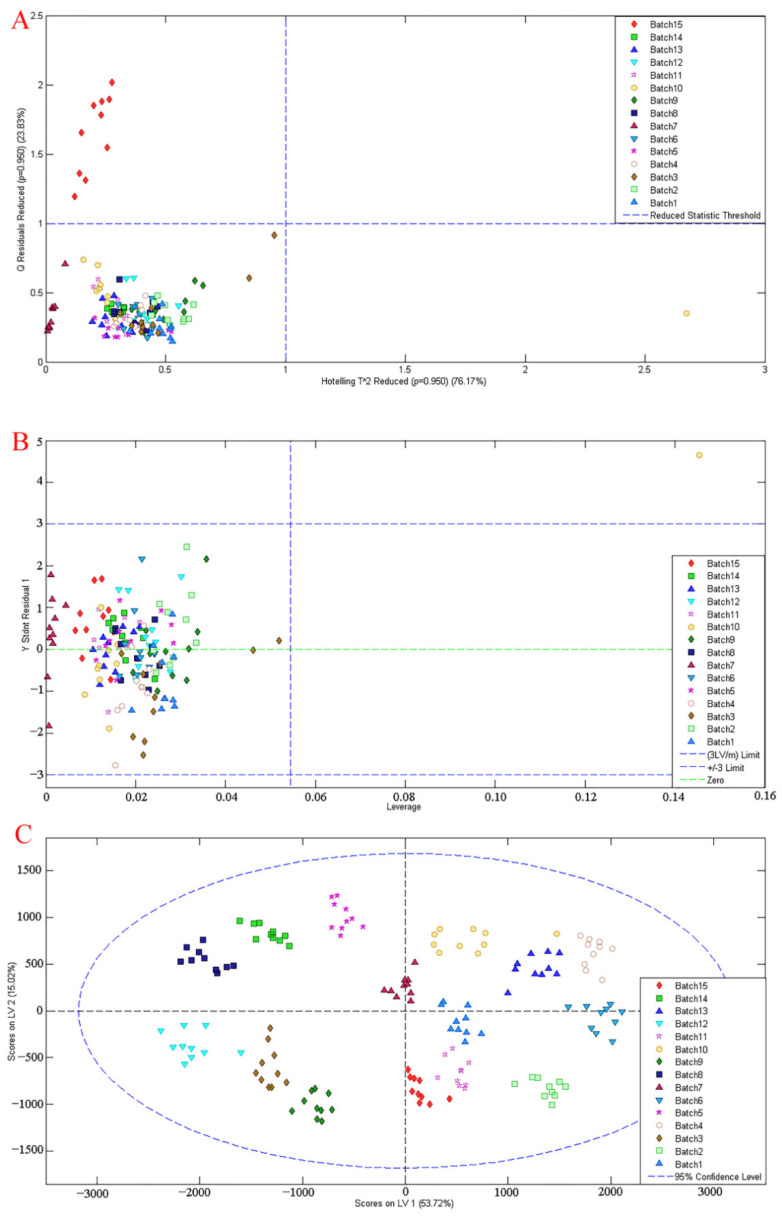
Data evaluation: (**A**) hotelling and Q-residuals plot; (**B**) Residuals vs. Leverage; (**C**) hotelling score. Different colors represent different API concentrations in Table 1.

**Figure 6:**
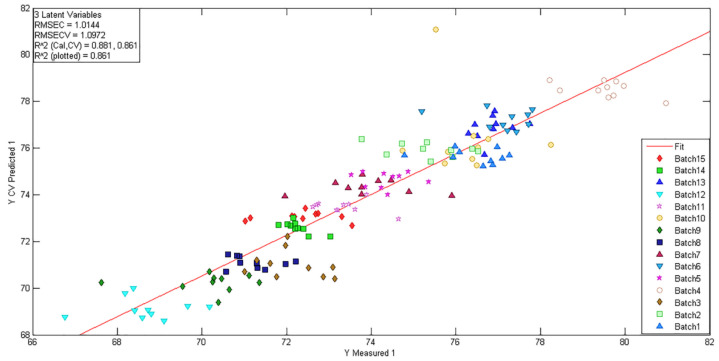
The plot of measured vs. predicted values (different colors represent different API concentrations in Table 1).

The scores of the model built using 15 levels are exhibited in Figure 5 and Figure 6. As shown in Figure 5, we assessed the degree of dispersion of the data from different perspectives, examining whether there were particularly extreme points in the data. Hotelling and Q-residuals are further model statistics that can be used to judge model performance and sample quality within a calibration sample set. The two statistics describe the similarities of samples within the calibration space. From Figure 5A, it can be seen that most of the samples sat within the reduced statistic threshold, but the red (samples of level 15 in Table 1) samples were outliers and sat away from the other samples. It may be that there were unknown compounds that produced noise interference during the mixing with API. The score of Residuals vs. Leverage in Figure 5B was used to judge whether there were extreme points, and we found one yellow point (one sample of level 10 in Table 1) that sat away from the other samples of the same level. In the manual tableting process, mixing time affects the similarity of tablets at the same concentration level. However, Figure 5C shows that 150 samples were all located within the 95% confidence level, and the samples of the same color were close to each other, so outliers did not need to be excluded and the model score was acceptable.

The RMSEC (1.0144), RMSECV (1.0972), and R^2^ (0.881, 0.861) values are shown in Figure 6. The closeness in values of RMSEC and RMSECV indicates that the model scores were acceptable. The 150 points were a bit scattered, which may have been caused by insufficient mixing of materials during the sample preparation process, and can be improved by increasing the mixing time to improve R^2^. The quality of the model should not be judged only by the model score, but also by the relative error between the predicted value and the true value during the validation process, which is mentioned in Section 2.1.3.

#### 2.1.3. Model Validation

**Figure 7:**
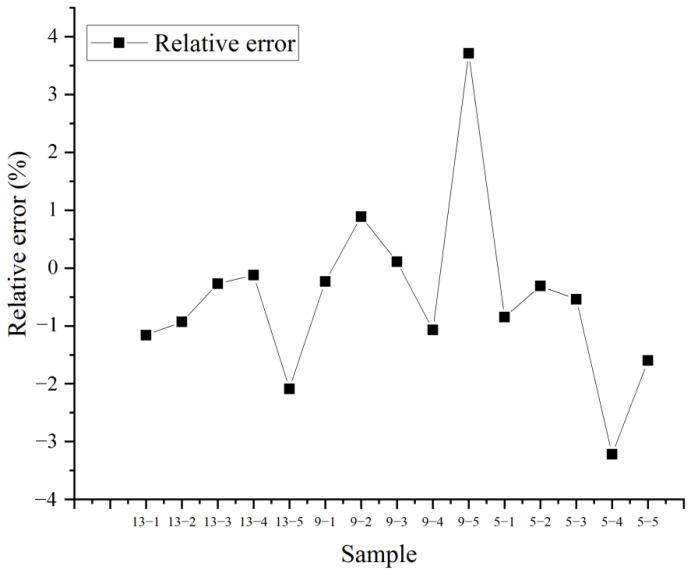
The relative error between HPLC and TRS.

In order to evaluate whether the model was established successfully, it was necessary to use the model to predict results for actual tablets. The established model was validated by taking the HPLC result as the true value and the TRS result as the predicted value. The established models with 15 levels were used to predict the contents of 15 samples (3 levels (• in Table 1) × 5 tablets) using TRS and HPLC, in order to validate the feasibility of the model. The results in Figure 7 show that the relative errors between TRS and HPLC were basically within 2%, which indicates that the model predicted the API content feasibly and accurately. Although there were three samples that exceeded 2%, due to the high concentration of API used in this study, the API and excipients may not have been uniformly mixed during the self-made tablet process, which may have caused difference in tablets. Tablets could be mixed by machine or over an increased mixing time to eliminate this difference. Additionally, as shown in Table 2, the linear regression equation y = 0.7672x + 16.79 (x means the content measured by HPLC, y means the content measured by TRS) was applied and the R^2^ was 0.9099. These results show that TRS guaranteed the accuracy and precision of the measurement with high speed and saved time.

### 2.2. Quantification of Marketed Paracetamol Tablets

The composition and proportions of the commercially available tablets are often different from those of the handmade tablets used to build the model. In order to prove the availability of the model built, four commercially available paracetamol tablets (Panadol, Anlipai, Guike, and Jinlu) were randomly selected (four brands × five tablets) and two tablets of each brand were scanned using TRS to obtain eight spectra (four brands × two tablets). We used the spectra in the model that had already been built to correct the model, so that each brand corresponded to a model which was used to predict the contents of other three tablets. The predicted contents were then compared with the results of HPLC (shown in Table 3).

**Figure 8:**
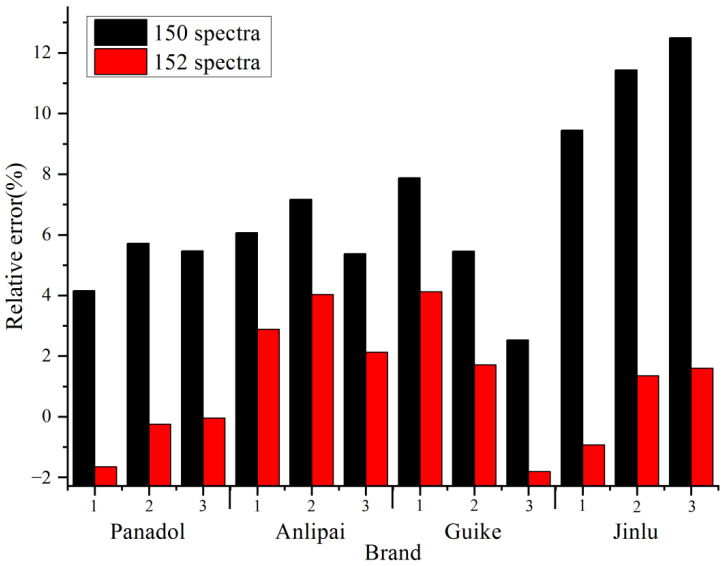
The comparison of relative error between the model built with 150 and 152 spectra.

As shown in Figure 8, in the process of quantifying the API contents of commercially available drugs, the models established using two tablets of the commercial drugs and 15 levels (152 spectra in total) were more accurate in predicting the content. Compared with the model built with only 15 levels (150 spectra in total), the relative error was greatly reduced. In the four corrected models, the relative errors of the content predicted by TRS were less than 5% compared with the results of HPLC. Because the compositions and proportions of the four branded tablets were different from the tablets made by hand, the relative error values in the prediction process were within the acceptable range, which shows that the model is suitable for determination of API content of commercially available paracetamol tablets. For different manufacturers, their tablets were used to correct the model and make it suitable for determination of the manufacturer’s paracetamol tablets, which makes the model widely applicable.

**Figure 9:**
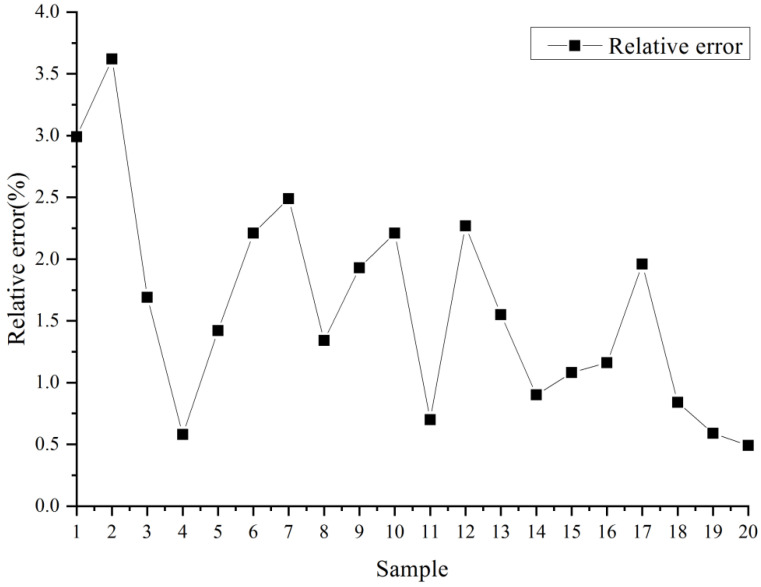
The relative error between HPLC and TRS.

Repeatability is one of the most important factors in quantitative assays using TRS. A total of 20 paracetamol tablets sold under the brand of Panadol (17.60 × 7.42 × 5.00 mm^3^, with white coating) was selected and the content was measured by TRS (shown in Table 4 and Figure 9). The RSD of the content measured by TRS was 0.86% and the relative error of the results between HPLC and TRS was basically within 4.00%. For the determination of the API content of 20 tablets, TRS was able to complete determination rapidly. Compared with HPLC, it greatly saves analysis time, and has good accuracy and repeatability.

## 3. Materials and Methods

### 3.1. Materials

The materials used were: TRS instrument (Agilent TRS 100, Wokingham, UK), high-performance liquid chromatograph (Thermo Ultimate 3000, Thermo Fisher Scientific, Waltham, MA, USA) with Chromeleon 7 software, acetaminophen (Anqiu Lu’an Pharmaceutical Co., Ltd., Weifang, China), pregelled starch (Kolorcon, Shanghai, China), calcium carbonate (Spectrum Chemical Manufacturing Corp., Shanghai, China), crospovidone (Anhui Sunhere Pharmaceutical Excipients Co., Ltd., Anhui, China), sodium propyl p-hydroxybenzoate (Xuzhou Donghe, Jiangsu, China), povidone K25 (BASF SE), alginic acid (Qingdao bright moon seaweed group CO., LTD., Qingdao, China), silica (Anhui Sunhere Pharmaceutical Excipients Co., Ltd., Anhui, China), magnesium stearate (Huzhou Zhanwang Pharmaceutical Co., Ltd., Huzhou, China), Panadol paracetamol tablets (17.60 × 7.42 × 5.00 mm^3^, with white coating, SK&F), Anlipai paracetamol tablets (17.10 × 7.86 × 5.40 mm^3^, Anhui Yongshengtang, Pharmaceutical Co., Ltd., Fuyang, China), Guike paracetamol tablets (diameter 12.06 mm, height 4.5 mm, Shijiazhuang Shi Huaxin Pharmaceutical Co., Ltd., Luancheng, China), Jinlu paracetamol tablets (diameter 12.06 mm, height 4.6 mm, Beijing Shuguang Pharmaceutical Co., Ltd., Beijing, China).

### 3.2. Preparation of Samples

According to the preparation instructions, the prescriptions were determined and powder mixtures were prepared according to DOE to make the samples. A total of 150 samples (15 levels × 10 tablets) were prepared according to the design shown in Table 1. Each powder with multiple ingredients of each level was enough to make 10 tablets. This allowed us to cover the whole calibration space while minimizing the number of samples to be prepared. The mixtures of API and excipients were mixed and compressed using a 17.5 × 7.5 × 5.5 mm^3^ flat surface tablet die in a DP30A single-punch tablet machine into 10 tablets per level. The tablets weighed on average ~660 mg with a range between 650 and 670 mg.

### 3.3. Experimental Conditions

#### 3.3.1. Transmission Raman Spectroscopy Conditions

The TRS spectra of API and mixture of excipients were collected using a TRS instrument and ContentQC software. The acquisition parameters employed a 4 mm laser illumination spot diameter at 0.5 W laser power with an M-type signal collector, and the acquisition time was 10.5 s (0.35 s × 30 accumulations). The processing method of spectra was derivative, multiplicative scatter correction, and mean center (DMM). All TRS spectra were recorded from 1700 cm^−1^ to 170 cm^−1^ and brought into the Solo software with the corresponding concentrations (%) to build a calibration model.

#### 3.3.2. Chromatographic Conditions of HPLC-UV

The API contents of the tablets were evaluated using an HPLC-UV method. The separation was carried out at 30 °C using an MGIIC18 250 mm × 4.6 mm × 5 μm column (Capcell pak, Shiseido, Japan), with a mobile phase containing methanol: water (1:3) at a flow rate of 1.5 mL min^−1^ for 6 min. Detection was carried out at 243 nm. Under these conditions, paracetamol had a retention time of 3.95 min. The results of API content measured by HPLC were brought into the model to replace the original contents in order to calibrate the model.

## 4. Conclusions

This study demonstrated the feasibility of quantifying the content of pharmaceutical tablets noninvasively using TRS. The model was established and optimized by changing the parameters, then used to measure the contents of four commercially available paracetamol tablets. For the quantification of active ingredients, TRS was found to be suitable for multivariate regression model development, resulting in models with increased predictive capacity. The method can greatly reduce the analysis period and sample consumption, achieve online analysis on the production line, and realize the quality control of drugs during the production process. The ability to yield spectrum-specific information and rapidly predict the content of API will unlock a range of new applications in pharmaceutical settings.

## Figures and Tables

**Table 1:** Formulation of tablets.

Sample	Mixture ^♦^ (mg)	Calcium Carbonate (mg)	Pregelled Starch (mg)	API (mg)	Total (mg)	API (%)
1 *	266.6	992.9	1125.9	7607.4	9992.8	76.13
2	299.2	1199.7	892.8	7591.4	9983.1	76.04
3 *	300.1	1198.0	1361.7	7122.7	9982.5	71.35
4 *	300.9	783.1	889.8	8009.9	9983.7	80.23
5 ^•^	299.4	783.1	1363.3	7560.5	10,006.3	75.56
6	379.0	990.4	790.1	7832.2	9991.7	78.39
7 *	378.6	989.5	1125.0	7500.5	9993.6	75.05
8	378.5	990.9	1464.6	7162.5	9996.5	71.65
9 ^•^	379.0	1288.5	1125.6	7195.3	9988.4	72.04
10	377.7	693.8	1126.0	7794.4	9991.9	78.01
11 *	457.5	1198.9	889.6	7454.0	10,000.0	74.54
12	457.6	1198.2	1362.2	6970.3	9988.3	69.78
13 ^•^	457.2	783.7	889.9	7862.2	9993.0	78.68
14 *	408.2	729.4	1271.3	6885.2	9294.1	74.08
15 *	490.1	990.3	1126.0	7400.5	10,006.9	73.95

* Indicates calibration samples, ^•^ indicates samples removed from calibration for use as independent validation samples, ^♦^ indicates the mixture of crospovidone (23.41%), sodium propyl p-hydroxybenzoate (2.34%), povidone k25 (9.93%), alginic acid (59.48%), silica (1.63%), and magnesium stearate (3.21%).

**Table 2:** The comparison of content measured between HPLC and TRS.

Sample	HPLC (%)	TRS (%)	Relative Error (%)
13-1	77.74	76.84	−1.16
13-2	77.34	76.62	−0.93
13-3	76.95	76.74	−0.27
13-4	76.87	76.78	−0.12
13-5	76.67	75.07	−2.09
9-1	70.46	70.30	−0.23
9-2	69.55	70.17	0.89
9-3	70.26	70.34	0.11
9-4	71.35	70.59	−1.07
9-5	67.62	70.13	3.71
5-1	74.24	73.61	−0.85
5-2	73.86	73.63	−0.31
5-3	74.67	74.27	−0.54
5-4	76.36	73.90	−3.22
5-5	74.40	73.21	−1.60
Linear regression equation	y = 0.7672x + 16.79		
R^2^	0.9099		

**Table 3:** The results of the model of 150 spectra and 152 spectra.

Brand		The Model of 150 Spectra ^1^			The Model of 152 Spectra ^2^		
		HPLC (%)	TRS (%)	Relative Error (%)	HPLC (%)	TRS (%)	Relative Error (%)
Panadol	1	82.30	85.44	3.68	82.30	80.67	−2.02
	2	80.82	85.44	5.72	80.82	80.62	−0.25
	3	80.82	85.24	5.47	80.82	80.78	−0.05
Anlipai	1	86.12	91.35	6.07	86.12	88.60	2.88
	2	85.39	91.52	7.18	85.39	88.82	4.02
	3	86.82	91.49	5.38	86.82	88.66	2.12
Guike	1	84.27	90.91	7.88	84.27	87.74	4.12
	2	86.02	90.72	5.46	86.02	87.49	1.71
	3	85.83	88.00	2.53	85.83	84.28	−1.81
Jinlu	1	80.34	87.93	9.45	80.34	79.59	−0.93
	2	79.87	89.00	11.43	79.87	80.95	1.35
	3	78.51	88.32	12.50	78.51	79.76	1.59

^1^ The model of 150 spectra was built with the 15 levels in Table 1; each level contains 10 handmade tablet spectra (150 spectra in total). ^2^ The model of 152 spectra was built on the basis of the model of 150 spectra, with each commercial brand model including two commercially available tablet spectra to correct the model (152 spectra in total). Each brand corresponded to its own exclusive model.

**Table 4:** The comparison of content measurements between HPLC and TRS.

Sample	HPLC (%)	TRS (%)	Relative Error (%)
1	79.81	82.20	2.99
2	79.45	82.33	3.62
3	80.60	81.96	1.69
4	81.19	81.66	0.58
5	81.86	83.02	1.42
6	81.00	82.79	2.21
7	80.72	82.77	2.54
8	81.23	82.32	1.34
9	81.41	82.98	1.93
10	80.89	82.68	2.21
11	82.38	82.96	0.70
12	80.74	82.57	2.27
13	81.35	82.61	1.55
14	81.92	82.66	0.90
15	81.40	82.28	1.08
16	81.56	82.51	1.16
17	80.58	82.16	1.96
18	81.31	81.99	0.84
19	81.55	82.03	0.59
20	81.72	82.12	0.49
RSD (%)	0.46	0.86	

## Data Availability

Not applicable.

## Sample Availability

Samples of the compounds are not available from the authors.
